# Calcium as a Key Player in Arrhythmogenic Cardiomyopathy: Adhesion Disorder or Intracellular Alteration?

**DOI:** 10.3390/ijms20163986

**Published:** 2019-08-16

**Authors:** Francesco Moccia, Francesco Lodola, Ilaria Stadiotti, Chiara Assunta Pilato, Milena Bellin, Stefano Carugo, Giulio Pompilio, Elena Sommariva, Angela Serena Maione

**Affiliations:** 1 Laboratory of General Physiology, Department of Biology and Biotechnology “L. Spallanzani”, University of Pavia, 27100 Pavia, Italy; 2 Center for Nano Science and Technology, Italian Institute of Technology, 20133 Milan, Italy; 3 Vascular Biology and Regenerative Medicine Unit, Centro Cardiologico Monzino IRCCS, 20138 Milan, Italy; 4 Department of Anatomy and Embryology, Leiden University Medical Center, Einthovenweg 20, 2333 ZC Leiden, The Netherlands; 5 Cardiology Unit, San Paolo Hospital, Department of Health Sciences, University of Milan, 20126 Milan, Italy; 6 Department of Clinical Sciences and Community Health, University of Milan, 20126 Milan, Italy

**Keywords:** arrhythmogenic cardiomyopathy, desmosomes, plakophilin-2, type 2 ryanodine receptors, phospholamban, Ca^2+^ sparks

## Abstract

Arrhythmogenic cardiomyopathy (ACM) is an inherited heart disease characterized by sudden death in young people and featured by fibro-adipose myocardium replacement, malignant arrhythmias, and heart failure. To date, no etiological therapies are available. Mutations in desmosomal genes cause abnormal mechanical coupling, trigger pro-apoptotic signaling pathways, and induce fibro-adipose replacement. Here, we discuss the hypothesis that the ACM causative mechanism involves a defect in the expression and/or activity of the cardiac Ca^2+^ handling machinery, focusing on the available data supporting this hypothesis. The Ca^2+^ toolkit is heavily remodeled in cardiomyocytes derived from a mouse model of ACM defective of the desmosomal protein plakophilin-2. Furthermore, ACM-related mutations were found in genes encoding for proteins involved in excitation‒contraction coupling, e.g., type 2 ryanodine receptor and phospholamban. As a consequence, the sarcoplasmic reticulum becomes more eager to release Ca^2+^, thereby inducing delayed afterdepolarizations and impairing cardiac contractility. These data are supported by preliminary observations from patient induced pluripotent stem-cell-derived cardiomyocytes. Assessing the involvement of Ca^2+^ signaling in the pathogenesis of ACM could be beneficial in the treatment of this life-threatening disease.

## 1. Introduction

The heart is a highly specialized machine that requires fine regulation: intracellular changes and cell-to-cell communication disturbances could destroy this balance. Depending on the type of cardiac arrhythmia, a series of events may occur: intracellular dysfunction‒tissue remodeling‒impaired cell‒cell connection–electrical dysfunction. Loss of cardiac tissue integrity could be both a cause and an effect of arrhythmic phenotype. A typical example is arrhythmogenic cardiomyopathy (ACM), a cardiac disorder in which mutations in proteins of the desmosome may be present, causing cell-to-cell discontinuity, arrhythmic events, and myocardial fibro-adipose substitution [1,2,3].

Intracellular calcium (Ca^2+^) signals drive excitation‒contraction (EC) coupling and are, therefore, necessary for the heart to effectively pump blood into the pulmonary artery and aorta. It is, therefore, not surprising that any defect in the Ca^2+^ cycling machinery or in the complex network of Ca^2+^-related proteins that maintain cardiac Ca^2+^ homeostasis under tight control may severely compromise cardiac function. Alterations or mutations in the Ca^2+^ handling machinery have been associated with a number of severe cardiac diseases, including cardiac hypertrophy, dilated cardiomyopathy, and heart failure [4,5], and several inherited arrhythmia syndromes (i.e., Timothy syndrome, Brugada syndrome, and early repolarization syndromes) [6,7,8].

Herein, we describe the emerging evidence in favor of Ca^2+^ dysregulation as a primary (or arrhythmia-causative) event in ACM. We discuss how the Ca^2+^ cycling machinery could be rewired into a pro-arrhythmogenic phenotype by some of the desmosomal mutations that are known to induce ACM by causing cardiac tissue discontinuity. Furthermore, we illustrate the effect of mutations in ACM causative genes that encode for crucial components of the Ca^2+^ handling system.

## 2. Calcium Signaling and Cardiac Arrhythmias: A Tight Connection

Cardiac contraction is triggered by a transient increase in intracellular Ca^2+^ concentration ([Ca^2+^]_i_) induced by membrane depolarization according to a process known as EC coupling [6,7,9]. Briefly, as the action potential, triggered by an influx of sodium (Na^+^) via the voltage-gated Na^+^ channels, propagates along the T-tubules, membrane depolarization opens CaV1.2, which is the pore-forming α-subunit of L-type voltage-gated Ca^2+^ channels (VGCCs), thereby causing the influx of Ca^2+^ into the dyadic junction, which separates the sarcolemma from the closely apposed (~15 nm) junctional sarcoplasmic reticulum (SR) [10] in ventricular myocytes [9]. Extracellular Ca^2+^ influx causes a large increase in local [Ca^2+^]_i_, which activates a cluster (7–20) of type 2 Ryanodine receptors (RYR2) in a process known as Ca^2+^-induced Ca^2+^ release (CICR) and gives rise to significant Ca^2+^ release events from the junctional SR (called Ca^2+^ sparks). The temporal summation of these local events by the propagating action potential results in a regenerative Ca^2+^ transient that is detected by troponin C to initiate the sliding of the thick (myosin) and thin (actin) filaments, cell shortening, and hence pressure development within each ventricle and ejection of blood into the circulation during cardiac systole [6,7,9]. Subsequently, [Ca^2+^]_i_ drops and mechanical force relaxes quickly to pre-systolic levels during cardiac diastole, which is essential to enable cardiac chambers to refill with blood. The increase in [Ca^2+^]_i_ is indeed short-lived as cytosolic Ca^2+^ is extruded across the plasma membrane by the Na^+^/Ca^2+^ exchanger (NCX1) and the plasmalemmal Ca^2+^-ATPase (PMCA) and sequestered back into the SR by the sarco-endoplasmic reticulum Ca^2+^ ATPase 2a (SERCA2a) [6,7,9]. The Ca^2+^ affinity of SERCA2a is regulated by the phosphoprotein phospholamban (PLN), which increases or reduces the pumping rate of SERCA2a depending on its phosphorylation state. In the dephosphorylated state, PLN binds to SERCA2a and inhibits the Ca^2+^ transport ability of the pump by reducing its apparent affinity for Ca^2+^. However, when PLN is phosphorylated, it dissociates from SERCA2a, thereby increasing the pump’s affinity for Ca^2+^ and favoring relaxation [11]. PLN presents two sites of phosphorylation: Ser16 for protein kinase A (PKA) and Thr17 for Ca^2+^/Calmodulin-dependent protein kinase II (CaMKII) and protein kinase B (AKT) [12]. Importantly, β-adrenergic stimulation exerts a profound impact on cardiac Ca^2+^ handling by modulating CaV1.2, RYR2 and SERCA2a activity. Accordingly, stimulation of β-adrenergic receptors, e.g., with isoproterenol, engages PKA and CaMKII. PKA, in turn, phosphorylates CaV1.2 and PLN, thereby increasing the amplitude of L-type voltage-gated Ca^2+^ currents and boosting SR Ca^2+^ uptake. Furthermore, Bovo and colleagues recently demonstrated that PKA-dependent phosphorylation also regulates RYR2-mediated SR Ca^2+^ release independently of PKA effects on SR Ca^2+^ load [13].

Evidence that has emerged over the last 20 years about inherited mutations in genes encoding for proteins involved in the regulation of Ca^2+^ homeostasis has clearly showed how defects in intracellular Ca^2+^ handling are associated with various forms of arrhythmogenic disorders, including catecholaminergic polymorphic ventricular tachycardia (CPVT), Brugada syndrome, Timothy syndrome, long and short QT syndrome [6,7,8]. These life-threatening diseases feature distinctive electrocardiogram patterns, ventricular arrhythmias, and a high risk of sudden cardiac death [6,7,8]. The major genes responsible for such inherited arrhythmias are *RYR2* (encoding for RYR2), *CASQ2* (encoding for calsequestrin-2), and *CACNA1C* (encoding for CaV1.2) [6,7,8].

RYR2 receptors cluster into functional Ca^2+^-release units presenting a finite open probability and are, therefore, able to mediate unsynchronized Ca^2+^ sparks even at diastole, when Ca^2+^ leaks out of the SR [9,14]. While the Ca^2+^ leak finely tunes the SR Ca^2+^ concentration, a pathological increase in the rate of Ca^2+^ efflux from the SR is detrimental to the heart as it can deplete the SR Ca^2+^ content, thereby reducing the amplitude of voltage-dependent Ca^2+^ transients and diminishing force production [9,14]. In addition, the increased SR Ca^2+^ leak during diastole can increase the frequency of spontaneous Ca^2+^ sparks, which can coalesce into a sub-sarcolemmal Ca^2+^ wave cleared by NCX1. This results in an untimely depolarizing (3 Na^+^ in: 1 Ca^2+^ out) inward current that triggers delayed afterdepolarizations (DADs) and induces ventricular arrhythmia [9,14]. An increase in RYR2-mediated Ca^2+^ leak has been observed in failing and aging hearts due to an increase in the extent of CaMKII-dependent phosphorylation [8,14]. Furthermore, a number of gain-of-function mutations in *RYR2* increases the SR Ca^2+^ leak in CPVT1, the autosomal dominant form of CPVT [15]. CPVT is an inherited arrhythmia disorder featuring polymorphic or bidirectional ventricular tachycardia; It arises in response to β-adrenergic stimulation during exercise or acute emotional stress and may lead to syncope and sudden cardiac death [7,8]. Arrhythmias in CPVT are induced by spontaneous RYR2-mediated Ca^2+^ release, which stimulates DADs to induce extrasystolic beats [8,15].

In turn, the recessive form of this pathology (CPVT2) has been associated with *CASQ2* mutations [16,17]. Calsequestrin-2 (CASQ2) is a moderate-affinity, high-capacity SR Ca^2+^ binding protein that maintains a reservoir of releasable Ca^2+^ in close proximity of the luminal mouth of RYR2 pore, while preserving low levels of free Ca^2+^ [8]. In addition, CASQ2 inhibits both Ca^2+^-induced and spontaneous RYR2 activation, thus acting as a negative regulator of SR Ca^2+^ release [18]. Consistently, a number of CPVT2-related mutations that increase CASQ2 proteolytic degradation, preventing its Ca^2+^-buffering activity or impairing its ability to modulate RYR2 opening, were found to induce spontaneous Ca^2+^ release, DADs, and, ultimately, ventricular arrhythmia during β-adrenergic stimulation [6,7,8].

Pro-arrhythmogenic mutations may also target CaV1.2, which is the primary determinant of L-type Ca^2+^ current (I_Ca,L_) in the ventricular myocardium and drives EC coupling by recruiting RYR2 through the CICR process. A missense mutation was reported at the cytosolic COOH-terminal of CaV1.2, resulting in impaired voltage-dependent inactivation in I_Ca,L_ and thereby causing an aberrant increase in [Ca^2+^]_i_ and DADs in Timothy syndrome [7]. This is a rare (< 30 patients around the world), multisystem disorder characterized by cardiac, hand/foot, facial, and neurodevelopmental features that can cause the patient’s death by inducing 2:1 atrioventricular block, QT prolongation in the ECG, and ventricular tachyarrhythmia [7]. Novel genetic alterations in *CACNA1C* were also described by Wemhöner and colleagues, causing typical long QT syndrome (LQTS) without any clinical features of Timothy syndrome and leading to a gain-of-function activity of the channel combining different mechanisms [19].

Conversely, a loss-of-function mutation in *CACNA1C* observed in 10‒15% of patients caused a dramatic reduction in CaV1.2-mediated Ca^2+^ entry that was associated with ST-segment elevation and increased risk of sudden cardiac death secondary to polymorphic ventricular fibrillation or tachycardia in Brugada syndrome [20]. Likewise, CaV1.2-mediated Ca^2+^ entry is dramatically reduced in short QT syndrome (SQTS), another rare inherited disease characterized by a short QT interval combined with life-threatening ventricular arrhythmias. The SQTS may arise as a consequence of three mutations that target both the pore-forming α subunit (i.e., *CACNA1C*) and the ancillary β (i.e., *CACNB2B*) and δ (i.e., *CACN2D1*) subunits [20,21].

In addition, other genes related to abnormal Ca^2+^-machinery functioning can lead to cardiac disorders. In particular, in the case of CPTV, three other genes have been linked to the pathology: CPVT3 to CPVT5. In the case of CPVT3, the mutation is in the TECRL gene encoding for the *trans*-2,3-enoyl-CoA reductase-like protein. Even though alteration in this gene seems to have overlapping clinical features of both LQTS and CPVT, studies in patient induced pluripotent stem-cell-derived cardiomyocytes (iPSC-CM) revealed smaller [Ca^2+^]_i_ transient amplitudes, lower SR Ca^2+^ stores, and elevated diastolic [Ca^2+^]_i_, consistent with the presence of a CPVT-related SR Ca^2+^ leak [22]. CPVT4, on the other hand, is caused by a heterozygous mutation in the calmodulin (CAM) gene (CALM1). In vitro assays have demonstrated that the pathogenic mechanism relies on altered Ca^2+^-binding and leads to a defective interaction between RYR2 and CAM [23]. Lastly, CPVT5 is caused by a homozygous or compound heterozygous mutation in the triadin gene (*TRDN*). Triadin is a transmembrane protein that forms a quaternary complex with *RYR2*, *CASQ2*, and junction proteins. In vivo expression of the mutant protein by viral transduction in triadin knockout mice led to fragmentation, a reduction in contacts between SR and T-Tubules, and an alteration in Ca^2+^ release units’ (CRUs) structure, rendering hearts more prone to ventricular arrhythmias [24].

## 3. Arrhythmogenic Cardiomyopathy

Arrhythmogenic cardiomyopathy (ACM) is a progressive heart disease characterized by fibrofatty substitution of the right (ARVC), left (ALVC), or both cardiac ventricles. Clinical manifestations include ventricular arrhythmias, impaired ventricular systolic function, heart failure, and sudden cardiac death. ACM is a genetic disorder caused by mutations in genes encoding for desmosomal proteins and non-desmosomal mutations, including genes coding for Ca^2+^ cycling regulators, growth factors, and structural proteins [25,26,27] (Table 1). A causative genetic alteration can be identified in the majority of cases [27], but different triggers, such as inflammation and excessive exercise, can worsen the ACM phenotype. However, treadmill exercise has been shown to partially rescue the remarkable dysregulation in gene expression observed in a mouse model of ACM carrying myocyte-specific desmoplakin haplo-insufficiency [28]. Because of its multiple phenotypes and the variability in the presentation, no single diagnostic test is available for ACM diagnosis. The diagnostic strategy consists of the collection of several parameters, such as genetic, electrocardiographic, arrhythmic, morphological, functional, and histopathologic abnormalities [29,30]. The population prevalence ranges from 1:1000 to 1:5000 and phenotypic expression is more common in males (2:1 to 3:1) [31]. Fibrofatty infiltration and progressive loss of ventricular myocardium represent the typical tissue remodeling in ACM. The infiltration has an epicardial to endocardial progression [32] and mainly involves the so-called ‘triangle of dysplasia’ (right ventricle inflow tract, outflow tract, and apex). The left ACM form is characterized by the involvement of the left ventricular wall with no alterations of right ventricle function [33,34], while the biventricular form involves both ventricles and is characterized by systolic impairment and biventricular dilation, with arrhythmic events that originate from either ventricle [34].

**Figure 1:**
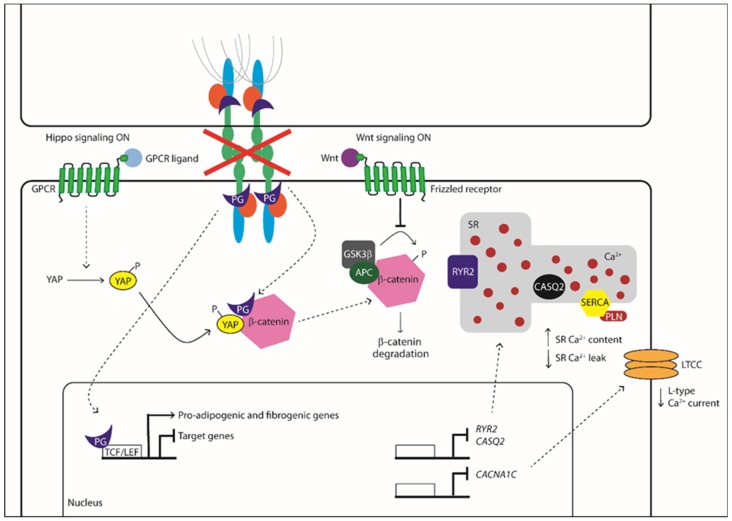
Molecular mechanisms associated with ACM pathogenesis. GPCR: G protein-coupled receptors, YAP: yes-associated protein, PG: Plakoglobin, TCF/LEF: T-cell factor/lymphoid enhancer-binding factor, Wnt: homologous wingless, APC: adenomatous polyposis coli, RYR2: type 2 ryanodine receptor, CASQ2: calsequestrin 2, CACNA1C: calcium voltage-gated channel subunit alpha1 C, SR: sarcoplasmic reticulum, SERCA: sarco-endoplasmic reticulum Ca^2+^ ATPase, PLN: phospholamban, LTCC: L-type calcium channel. (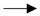 Activation/Phosphorylation, 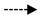 Translocation, 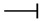 Inhibition).

The molecular mechanisms associated with ACM pathogenesis have been linked to dysregulation of the Wnt/β-catenin pathway, which leads to enhanced adipogenesis (Figure 1). The Wnt pathway is involved in cell proliferation, cell polarity, and cell fate determination during embryonic development and tissue homeostasis. The canonical Wnt signaling controls the developmental gene expression programs, acting through the transcriptional co-activator β-catenin. In the absence of Wnt ligands, the cytoplasmic β-catenin protein is constantly degraded; instead, Wnt ligands inhibit β-catenin degradation, leading to its accumulation in the cytoplasm and translocation into the nucleus, where it activates T cell/lymphoid-enhancing binding (Tcf/Lef) transcription factors. The β-catenin stabilization and translocation result in cell specification and differentiation [52]. The desmosomal protein plakoglobin (PG) also plays a key role in ACM pathogenesis because of its high functional and structural homology with β-catenin, including its increased localization in the nuclear compartment in ACM patients’ heart cells [53,54]. In the nucleus, PG may compete with β-catenin activity, leading to the suppression of Wnt signaling and the activation of the adipo-fibro-genetic transcription program [53].

It is important to note that the Wnt signaling does not work as an isolated pathway but is highly interconnected to other signaling pathways, including YAP/TAZ signaling, that have also been linked to ACM [55]. YAP and TAZ are two effectors of Hippo signaling, involved in cardiac development and regeneration [56]. YAP has been shown to act as an inhibitor of transforming growth factor beta (TGF-β) signaling by interacting with Smad7 and repressing the nuclear signaling [57]. Furthermore, during the Wnt inactive state, YAP and TAZ are associated with β-catenin into a destruction complex, thereby facilitating its ubiquitin-mediated-degradation. Wnt stimulation induces the YAP and TAZ dissociation from the destruction complex, resulting in β-catenin release [58] (Figure 1).

Based on their coexistence in the same macromolecular complex, the physical interaction between plakophilin-2 (PKP2) and the gap junction protein connexin 43 (Cx43) has been reported to highlight an electrical pathogenic mechanism for ACM [59]. Thereafter, it has been described that the voltage-gated Na^+^ channel Nav1.5 is also present in this interaction network, called a ‘connexome’ [29]. Notably, there is a reciprocal regulation between the components forming this structure. The Cx43 is responsible for expression [60,61] and cell membrane localization of Nav1.5 [62]. Furthermore, a loss of PKP2 causes Cx43 remodeling, leading to an alteration of intercalated disc structures [59] and reduced Nav1.5 functionality, which results in a decreased Na^+^ current and slower conduction velocity [63].

Besides contributing to desmosome formation and cell signaling [64], *PKP2* is also a regulator of RhoA activity in desmosomal plaque assembles [65]. RhoA is a member of the GTPases family and regulates gene transcription during cardiomyocyte differentiation through its downstream effector kinases, ROCKs [66]. RhoA activity is often enhanced in the myocardial tissue of ACM patients and correlates with PKP2/connexin43 regulation [67]. Moreover, in *PKP2*-mutated iPSC-CM, RhoA signaling is suppressed, leading to the overexpression of target genes such as serum response factor (SRF) and myocardin-related transcription factor (MRTF) and subsequent enhanced differentiation into adipocytes [66].

Increasing evidence indicates a correlation between the expression level of specific miRNAs and the regulation of cellular signaling involved in ACM pathogenesis. The miRNA profile of ACM and normal control heart samples showed that miR-21-5p and miR-135b are differently expressed in the two groups. MiR-21-5p has been linked to hypertrophic cardiomyopathy and fibrosis, dilated cardiomyopathy [68], and heart failure [69]. It was also demonstrated that miR-21-5p inhibition leads to reduced cardiomyocyte fibrosis and dysfunction in regulating the ERK/MAP kinase signaling pathway [70]. Both miR-21-5p and miR-135b are involved in Wnt and Hippo signaling pathways [71,72], and regulate the same target genes: *BMPR2*, associated with adipogenesis [73], and *TGFBR2*, which contributes to the extracellular matrix production. Transcriptome analysis of knockdown PKP2 HL-1 cells revealed that miR-184, whose expression is progressively reduced in cardiac myocytes during cardiac development, has a low expression in ACM. The downregulation of miR-184 is associated with the upregulation of its target gene, peroxisome proliferator-activated receptor gamma [74], leading to enhanced adipogenesis. Furthermore, the transcriptome characterization of ACM cardiac stromal cells showed that miR-29b and miR-1183 [75], already associated with ACM and other cardiac diseases, are upregulated compared to control cells. Notably, miR-29b plays a role in controlling the cardiac expression of collagen, fibrillin, and elastin genes [76], and also act as a regulator of Desmocollin 2 (DSC2) [77].

Despite all the abovementioned pathways having been linked to ACM, a full understanding of the molecular mechanisms leading to ACM pathogenesis is still missing, and other molecular players are likely involved. These include the dysregulation of the Ca^2+^ handling machinery that may occur as a consequence of mutations that affect desmosomal genes (i.e., *PKP2*) or some of the genes encoding for crucial components of the Ca^2+^ cycling machinery (i.e., *PLN* and *RYR2*).

## 4. Arrhythmogenic Cardiomyopathy as Adhesion Disorder: Ca^2+^-Dependent Desmosomes’ Stability

Cell to cell junctions are essential to confer stability to tissues, especially those undergoing continuous mechanical stretch, such as the heart and skin. The myocardium tissue integrity is based on desmosomes, anchoring cell junctions that are assembled in strong and highly specialized complexes. Loss or mutations of cell junctions are associated with human genetic diseases and ACM [39,53,78,79]. Desmosomes consist of specific cadherins, i.e., DSC2 and desmoglein-2 (DSG2), which act like a bridge to join the lateral edges of neighboring cells. The desmosomal cadherins bind proteins of the armadillo family, PG and PKP2, that are anchored to desmoplakin (DSP), the main intracellular component responsible for the adhesion to the intermediate filament network [80].

**Figure 2:**
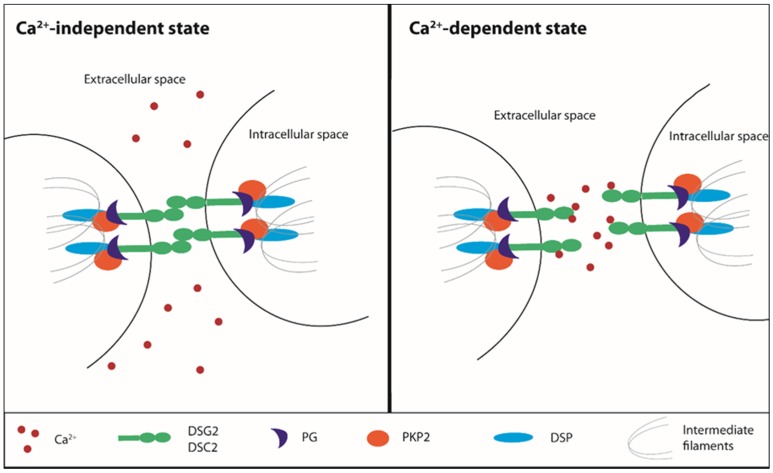
Ca^2+^-dependent desmosomes stability. Ca^2+^: calcium, DSG2: desmoglein 2, DSC2: desmocollin 2, PG: plakoglobin, PKP2: plakophilin 2, DSP: desmoplakin.

Desmosomes undergo a transition from hyper- to low-adhesion states depending on extracellular Ca^2+^ levels. The Ca^2+^-independent adhesive state of desmosomes is referred to as hyper-adhesion-state since it represents a higher-affinity and more stable binding ability both for desmosomes and adherens junctions. During tissue remodeling, wounding, and cell migration, a lower-affinity adhesive state is required and desmosomes adopt the Ca^2+^-dependent state, losing their organized structure [81,82,83] (Figure 2). This transition does not involve protein composition rearrangement, but it can influence the cadherin packing. Based on the nature of molecules underlying the desmosomal adhesion, both the formation and disruption are Ca^2+^-dependent mechanisms [84,85,86]. The desmosomal cadherins, DSC2 and DSG2, and the classical cadherin, E-cadherin, represent the Ca^2+^-dependent components of desmosomes and adherens junctions, respectively [87]. In vitro, the Ca^2+^-dependent mechanism is linked to the culture confluence state: in a confluent cell monolayer, desmosomes are Ca^2+^-independent until the confluence is destroyed by wounding the cell sheet, thereby resulting in the propagation of Ca^2+^ dependence through the whole monolayer [81].

## 5. Desmosomal Mutations and Arrhythmogenic Cardiomyopathy: Derangement of the Ca^2+^ Toolkit as a Consequence Rather than a Pathogenic Defect

The mechanism that induces the reduction of adhesion during tissue remodeling is not completely clear, but desmosomes can be totally internalized by cells. After the internalization, they are degraded or disassembled in order to recycle their component proteins [88,89]. During the Ca^2+^-dependence state, the desmosomes undergo a conformational rearrangement of the cadherin extracellular domains that induces a less organized structure and a more easily adhesive binding disruption [90]. The molecular basis of the adhesion between desmosomes involves the activation of protein kinase C (PKC) or inhibition of protein phosphatases [82,83]. The treatment with a low-Ca^2+^ medium induces loss of intercellular adhesion and the formation of half-desmosomes: the broken desmosomes are internalized by a PKC-dependent mechanism and transported to the centrosome, where they undergo proteasomal and lysosomal degradation [89]. The central role of PKCα is supported by experiments performed on a mouse model lacking or overexpressing this kinase. Both in absence and following inhibition of PKCα, the switch to Ca^2+^-dependence is blocked and hyperadhesive desmosomes lock the cells together, preventing cell motility and epithelial migration. On the contrary, in mice overexpressing a constitutively active PKCα, a rapid transition to a Ca^2+^ dependence state make desmosomes weakly adhesive, thus facilitating cell mobilization and promoting re-epithelialization [91].

Desmosomal mutations have been identified as a crucial determinant of ACM since the late 1980s [92]. A homozygous truncating mutation in the *JUP* gene, encoding for PG, was first identified through genetic linkage analysis as a typical syndromic form of right-sided ACM, known as Naxos disease [93]. Subsequently, genetic analysis revealed that another desmosomal gene, *DSP*, encoding for desmoplakin, harbored a homozygous truncating mutation in ACM in patients with a cardiocutaneous syndrome, named Carvajal syndrome [93,94]. These earlier discoveries sparked the search for causative mutations in additional desmosome genes in ACM patients and led to the identification of either missense or truncating mutations in the following desmosome genes: *PKP2* (encoding for plakophilin 2), *DSC2* (encoding desmocollin 2), and *DSG2* (encoding for desmoglein 2) (Table 1) [93]. It has been estimated that mutations in desmosomal genes are present in approximately half of ACM patients, with *PKP2* being the most commonly affected gene in adults [93]. The molecular mechanisms whereby *PKP2* mutations cause ACM are yet to be fully clarified, but no change in the Ca^2+^-sensitivity of the desmosomal junctions has been reported [93,95]. Conversely, desmosomal mutations can affect the electrical activity of the heart by compromising the expression, localization, and/or function of other components of the ‘connexome’, such as NaV1.5, the α subunit of voltage-gated Na^+^ channels, and Cx43 (see above). Intriguingly, a recent report demonstrated that the Ca^2+^ cycling machinery is defective in a conditional *Pkp2* knockout mouse model [95].

### PKP2

*PKP2,* encoding for plakophilin-2, is the most frequently mutated gene in ACM patients [37]. It has also been linked to other inherited cardiac arrhythmia syndromes, such as Brugada syndrome [96], idiopathic ventricular fibrillation, hypertrophic cardiomyopathy, and dilated cardiomyopathy, even if with a low frequency [97]. PKP2 is expressed in both myocytes and non-myocytes [54]. The main function of PKP2 is to guarantee mechanical stability during the desmosomal intermediate filament assembly required for cell-to-cell contact. PKP2 is part of the so-called ‘connexome’ [98]. Different studies highlighted the consequent novel role for PKP2 in the intracellular signaling regulation, electrophysiological and trafficking regulation, and the control of transcription processes [95].

Data from a cardiomyocyte-specific tamoxifen-activated *Pkp2* homozygous knockout (*Pkp2*-cKO) mouse model correlated the lack of PKP2 with transcriptional alteration of Ca^2+^ homeostasis [95]. This investigation reported the development of a cardiomyopathy of RV predominance that become evident at 21 days and then progressed into biventricular cardiomyopathy and HF. Transcriptome analysis showed that the transcripts of proteins involved in maintaining the intracellular Ca^2+^ concentration were downregulated in *Pkp2*-cKO hearts. The low level of transcripts relevant to Ca^2+^ cycling, i.e., *Ryr2*, *Ank2*, *Cacna1c*, and *Trdn*, accompanied a decreased expression of corresponding proteins and the impairment of the EC mechanism [95]. Accordingly, the downregulation of AnkB (encoded by *Ank2*) and triadin (encoded by *Trdn*), which contribute to maintaining the structural integrity of dyadic junctions [99,100], significantly reduced the distance between CaV1.2 and RYR2 [95]. In addition, *Pkp2*-cKO-derived cardiomyocytes showed decreased I_Ca,L_ density and a slower rate of current inactivation, which is consistent with the reduced expression of *Cacna1c* [95]. It should, however, be pointed out that, although the peak Ca^2+^ current was decreased, the total Ca^2+^ charge (i.e., the total amount of Ca^2+^ entering the cell upon membrane depolarization) was unaltered as compared to wild-type cardiomyocytes because of the slower inactivation rate [95]. Notably, the loss of PKP2 was also correlated with a reduction in the SR Ca^2+^ leak due to the downregulation of both *Ryr2* and *Casq2* [95] (Figure 1). As a consequence, the SR Ca^2+^ content was remarkably increased in *Pkp2*-cKO cardiomyocytes, which exhibited an increase in the amplitude and frequency of spontaneous Ca^2+^ release events (due to RYR2 sensitivity to intraluminal Ca^2+^ levels) and were therefore more prone to release SR Ca^2+^ during the EC coupling [95]. Accordingly, when *Pkp2*-cKO cardiomyocytes were paced at increasing rates, they displayed both early and delayed after-transients, which were sufficient to generate ventricular arrhythmogenic events during β-adrenergic stimulation with isoproterenol [95]. A more recent investigation focused on the early events driving the remodeling of the Ca^2+^ handling machinery in RV-derived PKP2-cKO (*Pkp2*-cKO-RV) cardiomyocytes isolated 14 days after tamoxifen injection [101], i.e., when cardiomyopathy was not evident yet. This report revealed an increase in RyR2-dependent Ca^2+^ release and RyR2-mediated Ca^2+^ sparks due to the remarkable elevation in the SR Ca^2+^ load that was caused by a Cx43-dependent increase in membrane permeability [101]. Notably, uncoupled Cx43 hemichannels may provide an alternative pathway for extracellular Ca^2+^ influx [102,103,104] and may, therefore, contribute to refilling the SR Ca^2+^ store in a SERCA2A-dependent manner. In addition, RyR2’s eagerness to release Ca^2+^ may be boosted by the observed phosphorylation in Thr2809, an amino acid residue near the consensus sequence for CaMKII and PKA [101]. These alterations were not detected in PKP2-cKO LV cardiomyocytes, thereby suggesting that this asymmetric dysregulation of the Ca^2+^ handling machinery precedes overt ultrastructural alterations and manifestations of ACM.

The relationship between PKP2 and Ca^2+^ machinery has also been highlighted by a recent bioinformatic approach that took advantage of a database containing transcriptomic information from human hearts, searching for coordinated transcription networks that are subjected to variations based on PKP2 abundance. The results were then validated with the information deriving from *Pkp2*-cKO murine hearts, thereby confirming the downregulation of *RYR2*, *ANK2*, and *CACNA1C* [105]. The results of the combined data supported the idea of a correlation between the PKP2 expression and the abundance of transcripts related to intracellular Ca^2+^ homeostasis. In this context, mathematical modeling confirmed that the PKP2-dependent downregulation of RYR2 and CASQ2 proteins is sufficient to cause the decrease in SR Ca^2+^ leak, which results in enhanced SR Ca^2+^ loading and EC coupling [95]. Accordingly, studies carried out on human iPSC-CM from a *PKP2*-mutated ACM patient showed that they present an abnormal Ca^2+^ handling capacity [106]. Of note, a recent investigation in iPSC-CM suggested that the Ca^2+^ handling machinery could also be affected by *DSG2* mutations. Accordingly, although the systolic and diastolic Ca^2+^ levels were similar, human ACM iPSC-CM exhibited spontaneous SR Ca^2+^ release and DADs in both the absence and the presence of β-adrenergic stimulation [107]. This investigation did not evaluate the molecular expression of Ca^2+^-related proteins, but further supports the notion that desmosomal mutations may affect the cardiac Ca^2+^ toolkit in ACM.

## 6. Non-Desmosomal Mutations and Altered Ca^2+^ Handling

As described in Section 2, mutations in Ca^2+^-related proteins may induce life-threatening inherited arrhythmogenic diseases, such as CPVT. It is, therefore, not surprising that, besides desmosomal mutations, ACM may also involve mutations that directly affect genes encoding for the Ca^2+^ cycling machinery, as suggested for RYR2 and PLN [12].

### 6.1. RYR2

Despite *RYR2* being mentioned in most ACM databases as associated with this disease, not all the scientific community agrees in considering it an ACM-causative gene. The phenotypic overlapping with CPVT leaves doubts about the proper clinical classification of patients in whom mutations were detected [108,109].

The *RYR2* gene encodes for a 565-kDa monomer that forms a homo-tetrameric structure associated with FK506-binding proteins that stabilize the channel in a closed state and are important for cooperative interactions among the RYR2 subunits. As previously mentioned, RYR2 is mostly known for its involvement in EC coupling, releasing Ca^2+^ from the SR and thus driving muscle contraction and heart beating [9,110,111]. Mutations in the *RYR2* gene have been reported in a family with an autosomal dominant form of ACM, associated with polymorphic ventricular tachycardia (induced by exercise stress testing) and a risk of sudden death. This condition was first described in 1988 and classified as arrhythmogenic right ventricular cardiomyopathy type 2 (ARVD2): a clinically different form because of its particular effort-induced ventricular arrhythmias, high penetrance, and 1:1 male: female ratio among the affected subjects [112]. Four missense mutations of *RYR2* were detected in ARVD2 patients [51] resulting in substitutions of highly conserved amino acids in the cytosolic portion of the molecule involving two regions also associated with malignant hyperthermia or central core disease and clustered in the corresponding skeletal muscle isoform, RYR1 [113]. The mutations occur in a regulatory domain of RYR2 known to be involved in the interaction with FKBP12.6 and therefore in the stabilization of a tetrameric structure [114]. A functional characterization of these mutations is yet to be provided at the single-channel level, but the R176Q substitution responsible for ARVD2 corresponds to the Arg163Cys substitution reported in RYR1, which dramatically increases its sensitivity to halothane in malignant hyperthermia [115]. It is, therefore, predictable that the *RYR2* mutations observed in ARVD2 patients enhance Ca^2+^ leakage and increase spontaneous SR Ca^2+^ release, thereby leading to DADs and ventricular arrhythmia [51]. In agreement with this hypothesis, transgenic mice carrying the R176Q mutation underwent ventricular tachycardia, which was associated with the appearance of spontaneous SR Ca^2+^ release events in both paced and non-paced isolated cardiomyocytes [116]. Notably, a recent structural investigation suggested that the R176Q mutation interferes with the Ca^2+^-induced molecular rearrangement of *RYR2*, thereby increasing the fractional Ca^2+^ release [117]. Besides being pro-arrhythmogenic, these mutations could promote mitochondrial Ca^2+^ overload and stimulate apoptosis [51], which has actually been observed in ARVD [118].

Six further rare missense variants have been identified in *RYR2*, mapping the locus to chromosome 1q42‒q43 [50,51] in four independent families with the same clinical manifestations and close to CPVT based on exercise-induced ventricular arrhythmias, high penetrance, and a 1:1 sex ratio. The described variants affect different *RYR2* domains [119]. Several mutations were found in the cytoplasmic region of the protein (NH2-terminal), whose alterations are responsible for the gain of function of RYR2 and are associated with hypertrophic cardiomyopathy and unexplained sudden cardiac death, as described elsewhere [120,121,122]. Missense variants mapped to the transmembrane domain of the protein and involved in the pore formation of the Ca^2+^ channel have been associated so far with CPVT or sudden death [119].

Two common single nucleotide polymorphisms in exon 37 of the human *RYR2* gene, causing non-conservative amino acid exchanges, i.e., G1885E and G1886S, have been associated with arrhythmogenic right ventricular cardiomyopathy (ARVC) [123]. These polymorphisms, found in a subgroup of patients in whom no known *RYR2* mutations have been detected, are responsible for creating a putative protein kinase C phosphorylation site known to be involved in cardiac contractility and propensity to heart failure [124]. Genetic analysis and single-channel measurement of *RYR2* activity revealed that the combination of these polymorphisms in a single individual led to a leaky SR channel under diastolic conditions, thereby triggering arrhythmia and sudden cardiac death.

### 6.2. PLN

PLN is one of the main regulators of Ca^2+^ cycling and an effector of β-adrenergic stimulation. PLN is sensitive to PKA-dependent phosphorylation and PLN expression levels and its phosphorylation state is a key to fine-tuning SERCA activity. The de-phosphorylated PLN interacts with SERCA, resulting in a low Ca^2+^ affinity state that limits Ca^2+^ sequestration into SR lumen; when it is phosphorylated, the inhibitory activity on SERCA is abolished and the affinity of SERCA to Ca^2+^ is restored, so that a larger amount of Ca^2+^ is pumped back into the SR [9].Therefore, PLN is a crucial modulator of SR Ca^2+^ content and the major determinant of cardiac contractility and relaxation, depending on its phosphorylation levels: increased phosphorylation improves contractility; Vice versa, the de-phosphorylation state promotes heart failure [125].

Mutations in the human *PLN* gene were firstly associated with dilated cardiomyopathy (DCM) [126] Patients, but have also been reported for patients diagnosed with ACM [45], as for the *PLNArg14Del* mutation. This is a deletion of Arg-14 in the coding region and has been identified in a family in which heterozygous carriers exhibited inherited DCM and died by middle age. Notably, a mouse model with cardiac-specific expression of the heterozygous *PLNArg14Del* mutant recapitulates the human phenotype, such as depressed cardiac function, fibrosis, and premature death [126]. At molecular level, the *PLNArg14Del* mutation prevents the phosphorylated PLN from relieving SERCA inhibition, thereby causing a reduction in SR Ca^2+^ concentration [12]. Accordingly, when SERCA2 was co-expressed with the *PLNArg14Del* mutant in HEK-293 cells, Ca^2+^ uptake into microsomal vesicles was severely impaired [126]. Likewise, SERCA2a-mediated Ca^2+^ transport in cardiac homogenates isolated from transgenic mice expressing the *PLNArg14Del* mutation was impaired as compared to wild-type samples [126]. Immunohistochemistry on samples derived from patients clinically diagnosed with DCM or ACM and carrying the *PLNArg14Del* mutation further revealed that plakoglobin is downregulated at cell junctions, mostly in ACM samples [45]. Moreover, a comparative analysis performed on myocardial tissue from ACM and DCM patients showed that, in ACM patients, *PLN* total mRNA, total and phosphorylated protein levels are significantly increased compared to healthy controls and patients with DCM [127]. This finding suggests a role of PLN in the pathogenesis of ARVC and indicates the existence of overlapping pathways with DCM [45].

The pathological mechanisms of the PLN mutation underlying ACM are still unclear, but, in general, the *PLNArg14Del* mutation is likely to result in reduced SR Ca^2+^ content. In agreement with this hypothesis, a recent investigation revealed that the intracellular Ca^2+^ dynamics were perturbed in human iPSC-CM deriving from a patient affected by DCM and bearing the *PLNArg14Del* mutation [128]. Indeed, although the releasable SR Ca^2+^ pool was significantly reduced, there was an increase in the diastolic Ca^2+^ levels, beating rate, and frequency of irregular Ca^2+^ waves [128]. Interestingly, when the *PLNArg14Del* mutant was introduced into a *PLN* null mouse model, it did not associate with SERCA2a, but translocated to the plasma membrane to physically interact with the Na^+^/K^+^ ATPase [129]. As a consequence, cardiac contractility became insensitive to β-adrenergic stimulation [126]. The causative relationship between the *PLNArg14Del* mutation and ACM could be better interpreted by considering another DCM-associated *PLN* mutation, i.e., R25C [130]. This mutation renders the heart susceptible to ventricular arrhythmia because of a dramatic remodeling of the Ca^2+^ cycling machinery. Indeed, when *R25C*-*PLN* was overexpressed in rat adult cardiomyocytes, it suppressed SERCA2a activity, thereby reducing SR Ca^2+^ content and the amplitude of voltage-dependent Ca^2+^ transients, with impairment of contractile activity following [130]. The resulting increase in diastolic Ca^2+^ levels was found to recruit CaMKII, which in turn phosphorylated RYR2 to induce an increase in SR Ca^2+^ leakage, spontaneous Ca^2+^ sparks, and resultant arrhythmias [130]. Compared to desmosomal mutation carriers, *PLN* mutation carriers more often showed T-wave inversion and are associated with additional left ventricle (LV) involvement and low-voltage electrocardiogram. However, as reported for desmosomal mutation carriers, *PLN* mutation carriers also showed RV disease, fibrofatty replacement, and arrhythmogenic phenotype [45,131]. In addition, PLN mutation carriers are unlikely to show downregulation of synapse-associated protein 97, which is commonly reduced in the ventricular sarcomeres of ACM patients, while they do not present redistribution of glycogen synthase kinase-3 beta to the intercalated disks, as reported in a desmosomal ACM cohort [132].

## 7. Conclusions

ACM is classified as a genetically determined cardiac disease that is clinically characterized by arrhythmic events. Different pathways have been associated with ACM and were mostly correlated with enhanced adipogenesis and electrical dysfunction. Despite the identification of different molecular mechanisms, a full understanding of the ACM pathogenesis is still lacking. Ca^2+^ dysregulation can be considered as a possible causative event based on its involvement in the cells’ adhesive property and its pro-arrhythmic ability. The desmosomes’ adhesive state is Ca^2+^-dependent and their structural stability consists of a PKC-mediated mechanism. Moreover, defects in the Ca^2+^ cycling machinery, also identified among the ACM-causative genetic mutations, are responsible for dysregulated Ca^2+^ homeostasis, leading to cardiac arrhythmia. Of note, a loss of *PKP2*, most frequently mutated in ACM patients, correlates with an alteration of Ca^2+^ machinery [95]. These observations raise the possibility that the ACM-causative mechanism may involve a defect in the expression and/or activity of the cardiac Ca^2+^ handling machinery, not only limited to myocytes, ultimately leading to arrhythmias, but also in the stromal compartment, which is actively involved in the pathological remodeling [54].

To date, pharmacological treatments generically targeting Ca^2+^ homeostasis, such as calcium antagonists and flecainide, are used to treat arrhythmias. However, confirming the involvement of Ca^2+^ signaling and understanding the molecular underpinnings of Ca^2+^ signaling in the pathogenesis of ACM could be pivotal to finding targeted therapies (cell-specific and/or molecule-specific) that could be beneficial for the treatment of this life-threatening disease.

## Figures and Tables

**Table 1:** Lists of genes involved in ACM pathogenesis.

	Gene	Coding Protein	Locus	Reference
desmosomal mutations	*JUP*	Junction Plakoglobin	17q21.2	[35]
	*DSP*	Desmoplakin	6p24.3	[36]
	*PKP2*	Plakophilin 2	12p11.21	[37]
	*DSG2*	Desmoglein 2	18q12.1	[38,39]
	*DSC2*	Desmocollin 2	18q12.1	[40]
non-desmosomal mutations	*TMEM43*	Transmembrane protein 43	3p25.1	[41]
	*LMNA*	Lamin A/C	1q22	[42]
	*DES*	Desmin	2q35	[43]
	*CTNNA3*	Alpha-T-catenin	10q21.3	[44]
	*PLN*	Phospholamban	6q22.31	[45]
	*TGFB3*	Transforming growth factor-3	14q24.3	[27,46]
	*TTN*	Titin	2q31.2	[47]
	*SCN5A*	Sodium voltage gated channel alpha subunit 5 (NaV 1.5)	3p22.2	[48]
	*CDH2*	Cadherin C	18q12.1	[49]
	*RYR2*	Type 2 ryanodine receptor	1q42-q43	[50,51]
